# The Effect of Intra-carpal Kirschner Wire Augmentation in Screw Fixation of Scaphoid – A Retrospective Cohort Study

**DOI:** 10.5704/MOJ.2011.016

**Published:** 2020-11

**Authors:** MQH Leow, SR Chung, SC Tay

**Affiliations:** 1 Biomechanics Laboratory, Singapore General Hospital, Singapore; 2 Department of Hand and Reconstructive Microsurgery, Singapore General Hospital, Singapore

**Keywords:** scaphoid fracture non-union, intracarpal kirschner wire, intramedullary screw, augmented fixation

## Abstract

**Introduction:**

Scaphoid fractures are most often treated with a single headless compression screw. However, intercarpal Kirschner wire (K-wire) might be added to improve stability and fracture outcomes. This study will determine if there is a difference in treatment outcome (union rate and time to union) between scaphoid fracture fixations using a single headless compression screw with and without augmentation using a intracarpal intramedullary K-wire.

**Material and Methods:**

We conducted a retrospective review of patients who underwent surgery for isolated scaphoid fractures over a 15 years period from December 2000 to December 2015. Only patients who underwent open surgery with bone grafting were included. They were divided into a group treated with a single screw fixation, and another group treated with screw and K-wire fixations.

**Results:**

Forty-four (58.7%) patients had single screw fixation and 31 (41.3%) had screw augmented with K-wire fixation. The overall union rate was 88.0%, with an overall mean time to union of 5.3 months. There was no difference in union rate (p=0.84) and time to union (p=0.66) between the single screw group and combined screw and K-wire group. Univariate analysis found that older age (t=-2.11, p=0.04) had a significant effect on union rate. Regression model showed that age had a significant effect on months to union.

**Conclusion:**

In open fixation of scaphoid fractures with compression screw and bone grafting, union rate and time to union is comparable whether or not screw fixation was augmented with an intracarpal K-wire. There was no increased risk of complications associated with augmented screw. Age of patient affected time to union and union rate.

## Introduction

The scaphoid is the most frequently fractured carpal bone, and accounts for 50% to 80% of all carpal fractures in young people^1^. The incidence of scaphoid fracture varies between 29 to 43 per 100,000 each year in the general population^2,3^, and can be as high as 121 per 100,000 in the military population^4^. Scaphoid fractures commonly occur due to a fall on outstretched hand with a hyperextended wrist. It is commonest in young men aged 15 to 29, who also have the highest incidence of non-union, and longest time to union^5^. A majority (64%) of scaphoid fractures occur at the waist, and a small percentage (4.6%) occur at the proximal pole^6^. Although most scaphoid fractures heal uneventfully, 5%–10% of scaphoid fractures develop non-union^7-9^. Common risks for non-union include displaced scaphoid fractures, proximal pole fractures, and avascular necrosis (AVN)^1,9-11^.

Scaphoid fractures can be treated conservatively or surgically^12^. Surgical fixation can be performed with headless compression screws, Kirschner wires (K-wires), and plates^13-17^. However, they are now most often treated with a single headless compression screw^14,18,19^.

With cannulated screw fixation, K-wires can be used to augment screw fixation of the scaphoid. There have been two papers published on augmenting scaphoid screw fixations with K-wires, but they are intercarpal, not intracarpal. Dodds *et al*^20^. performed a biomechanical study and found that an intercarpal K-wire (scaphocapitate) added more stability to a long screw fixation of scaphoid but not significantly. Allon *et al*^21^. believed that stabilising the construct after screw fixation of scaphoid with intercarpal scapho-capitate, and sometimes scapho-lunate K-wires, may improve union rate. However, they found no significant difference in union rate, time to union, complication rate, and post-operative range of motion. They concluded that intercarpal K-wires can be safely used to augment scaphoid screw fixation^21^.

It is not known if augmenting scaphoid screw fixation with intracarpal intramedullary K-wires would improve stability and improve union rate, or the additional drilling and presence of additional hardware would affect fracture union. As we could not find any papers looking at the effect of intracarpal K-wires being used to augment scaphoid screw fixation, we decided to look at our cases to evaluate if this would have any effect on fracture outcomes. As such, we performed an audit on all open scaphoid headless compression screw fixations with bone graft, to determine if there is a difference in fracture outcome (union rate and time to union).

## Materials and Methods

The study was approved by the hospital’s ethics committee (CIRB, 2012/870/D). A retrospective review of patients who underwent surgery for isolated scaphoid fractures over a 15-year period from December 2000 to December 2015 were identified using hospital records. There were a total of 136 patients of which 93 had open surgery, and 43 minimally invasive surgery (eg. arthroscopic, percutaneous). In the open surgery group, 87 had bone grafting. We excluded 6 patients, with double screw fixation and 6 patients with avascular necrosis from this group of 87 patients, giving a final study sample size of 75 patients.

All data extracted from patient charts and electronic records were recorded on a standardised form in Microsoft Excel. Patients’ demographic data, radiographs, operative records, and clinical data were obtained. Post-operative radiographs were typically performed weekly or bi-weekly for the first six weeks, and monthly thereafter until the fracture had united radiographically. Fracture union was identified based on the presence of trabecular bridging across fracture site in at least two scaphoid views with no gaps seen in any of the radiographic views^22,23^. Post-operative data was based on patients’ last documented visit.

Descriptive statistics were used to describe the participants’ socio-demographic characteristics and clinical data at baseline. For continuous data, mean and standard deviation were calculated. For categorical data, counts and percentages were used. Independent t-test was used to compare continuous variables between the two groups. Chi square and Fisher’s exact test (if count less than 4) were for categorical outcome variables. This was to check for any differences in socio-demographic data between both groups at baseline. Chi square test and odds ratio were used to compare union rate, which was a categorical data. T-test was used to compare time to union, which was a continuous data.

Univariate analysis was performed on the dataset to identify potential factors that could affect union rate and time to union, and the significant factors included in the regression model analysis for months to union. For the univariate analysis, Pearson correlation was used to compare two continuous variable, ANOVA test was used to compare three or more groups with continuous variables, t-test was used to compare two groups with continuous variables, and chi-square test was used to compare between two categorical data. A p-value of <0.05 was considered statistically significant.

## Results

There were 44 (58.7%) patients with single screw fixation (Fig. 1), and 31 (41.3%) patients with augmented screw with intracarpal K-wire fixation (Fig. 2). There were no differences in the demographic, radiographic, and operative data between both groups (Table I).

**Table I:** Clinical, radiographic, and operative data

	Single screw (n=44)	Combined screw and K-wire (n=31)	Statistic	p-value
**Clinical**				
Age (years)	25.0 (6.6)	28.6 (11.8)	t = -1.69	0.10
Male	44 (100.0)	29 (93.5)	Fisher = 0.17	0.09
Smoking	8 (18.2)	5 (16.1)	χ^2^ = 0.05	0.82
Dominant hand	18 (40.9)	16 (51.6)	χ^2^ = 0.84	0.36
Mechanism of injury			χ^2^ = 3.00	0.39
Falls	20 (45.5)	19 (61.3)		
Sports	15 (34.1)	7 (22.6)		
Road traffic accident	9 (20.5)	5 (16.1)		
**Radiographic**				
Proximal pole	17 (38.6)	18 (58.1)	Fisher = 0.69	0.49
Waist	27 (61.4)	18 (58.1)		
Distal	0 (0.0)	1 (3.2)		
DISI deformity	10 (22.7)	6 (19.4)	χ^2^ = 0.12	0.73
**operative**				
Time to surgery:				
≤3 weeks	3 (6.8)	3 (9.7)	Fisher = 0.69	0.65
>3 weeks	41 (93.2)	28 (90.3)		
≤6 months	27 (61.4)	18 (58.1)	χ^2^ = 0.08	0.77
>6 month	17 (38.6)	13 (41.9)		
≤12 months	36 (81.8)	24 (77.4)	χ^2^ = 0.22	0.64
>12 months	8 (18.2)	7 (22.6)		
Bone graft source			Fisher = 0.37	0.35
• Distal radius	16 (36.4)	6(19.4)		
• Iliac crest	4 (9.1)	2 (6.5)		
• Olecranon	24 (54.5)	20 (64.5)		
Vascularised bone graft	3 (6.8%)	2 (6.5%)	Fisher=0.64	0.38

The overall union rate was 88.0%, with an overall mean time to union of 5.3 months. The odds ratio for recovery using combined screw and K-wire was 0.87. The union rate using single screw was 88.6%, and 87.1.% for combined screw and K-wire. Mean time to union for single screw was 5.2 months, and 5.7 months for combined screw and K-wire. There was no statistical difference in union rate and time to union between the single screw group, and augmented screw group.

Age had a significant effect on union rate using univariate analysis (t=-2.11, p=0.04). Patients who had scaphoid union were older (mean=27.2, SD=1.2), compared to those who had non-union (mean=20.4, SD=0.8). Only age was significant in the regression model with respect to time to union (t=6.64, p=<0.001) (Table II).

**Table II:** Regression analysis for months to union

Predictor	Standardised β	t	p-value
Surgery method^a^	0.81	0.68	0.50
Age	0.17	6.64	<0.001

^a^Surgery method refers to single screw Kirschner Wire Augmentation. Adjusted R2=0.55, F=40.77. Model is significant p=<0.001

There was no significant difference in post-operative outcomes between both groups. Patients returned to work after 1.3 months and 1.7 months in the single screw group, and the augmented screw group, respectively (p=0.35). The follow-up duration in the single screw group and the augmented screw group was 12.7 months and 17.2 months, respectively, but the difference was not significant (p=0.17). Grip strength (p=0.86), wrist flexion (p=0.99), and wrist extension (p=0.34) were similar between both groups (Table III).

**Table III:** Post-operative outcomes

	Single screw	Combined screw and K-wire	t-test	p-value
Return to work, mean months (SD)	1.3 (1.0)	1.7(2.1)	t = -0.94	0.35
Follow-up, mean months (SD)	12.7 (13.9)	17.2 (13.2)	t = -1.39	0.17
Grip strength (% of uninjured)	85.5 (18.3)	84.5 (16.4)	t = 0.17	0.86
Wrist flexion (degrees)	63.9 (15.2)	63.8 (15.9)	t = 0.01	0.99
Wrist extension (degrees)	58.9 (14.9)	62.0 (11.3)	t = -0.96	0.34

Three patients in the single screw group had complications (7.3%). One patient had screw protrusion which required a secondary procedure to remove the screw, one patient had the screw displacement and required revision surgery, and one patient had hypertrophic scarring of the wound which was treated conservatively. In the augmented screw group, two had revision surgery due to the persistent non-union (6.5%). Ten out of 31 (32.3%) patients in the combined screw and K-wire group underwent removal of K-wire after the scaphoid had healed.

## Discussion

Fixations with K-wires or headless screws are well established methods of fixation of scaphoid fractures. Proponents of K-wire fixation often utilise multiple K-wires^24^, whereas screw fixations often involve the placement of one screw^25^. Double screw fixation has been reported but the numbers are low^26,27^. In our own centre, the rate of double screw fixation was 4.4% (6/136 patients).

Fracture site stability is important for scaphoid healing. In his surgical protocol, Slade *et al*^28^ would insert a second mini-screw into the scaphoid if he deemed the fixation to be insufficiently stable. If that is not possible due to insufficient space in the scaphoid or poor bone quality, he would insert an intercarpal K-wire or mini-screw from the distal scaphoid into the capitate to reduce micro-motion at the fracture site. He would keep this intercarpal fixation until the fracture has healed before removing at about 3-6 months. The use of an intercarpal K-wire as an addition to the rigid construct afforded by headless compression screws in scaphoid fractures has been investigated both in the laboratory and in the clinical setting^20,21^. However, both studies were not able to conclusively prove that the additional K-wire constructs improve biomechanical stability or improve union rate.

Despite the prevalent use of K-wires during scaphoid screw fixation, the results of augmenting screw fixations of scaphoid with intracarpal K-wires, has never been specifically investigated. It is not known if these intracarpal intramedullary K-wires which cross the fracture site with the screw would improve stability and improve union rate, or would the additional drilling and presence of additional hardware affect fracture union.

In our centre, 41% (31/75) of open scaphoid screw fixation with bone graft had intracarpal intramedullary K-wires. This is a sizeable proportion which had this augmented fixation. A 1mm K-wire is inserted before screw insertion. It is then bent and cut just under the skin as they are part of the fixation construct and need to be kept until fracture healing. As these K-wires do not cross any carpal joints, wrist mobilisation is not restricted. Intercarpal K-wires might need protection lest the wire break in the joint from motion. As such, final functional outcome may also be affected if intercarpal K-wires are kept for too long. Evidence shows that if the wires are removed early at six weeks, there would be no significant effect on final wrist motion^21^. However, it is not clear what functional outcome, and possible complications would ensue if they are kept for as long as six months^28^. Additional, intercarpal K-wires may potentially cause permanent damage to the midcarpal joint cartilage. However, intracarpal intramedullary K-wires may reduce the amount of bone interface available for healing at the fracture site^29^. Intercarpal K-wires, on the other hand, generally do not cross the fracture site and thus, the additional K-wire should have no direct interference with fracture healing.

Our union rate for the single screw, and augmented screw groups were 89% and 87%, respectively. Published systematic reviews have reported union rates of 80% to 88%, and 84% to 92% for internal fixation with non-vascularised bone grafts and vascularised bone grafts, respectively^30-32^. Our union rate is comparable with what is published in contemporary literature.

Our results show that the addition of intracarpal K-wire does not improve the union rate or reduce the time to union. However, we acknowledge that this is a retrospective study and we believe a prospective study could be done to validate this outcome.

Our study did not show any increased in the risk of complications with the additional intracarpal K-wire, other than the fact that patients may require a secondary procedure to remove it. This may require a slightly longer period of follow-up although the difference was not found to be statistically significant in our study. We think the presence of hardware outside the bone may cause these patients to need a slightly longer time off work, but again, in our study the difference in time off work between the groups was not statistically significant. It should also be noted that 61% (19/31 of the patients) of them did not have their intramedullary K-wire removed. Those who had the K-wire removed could probably feel the bent tip of the wire under the skin.

Our study found that age was a significant factor affecting the rate of union in scaphoid screw fixations with bone grafting, where younger patients would have slightly lower union rates. This is quite surprising as most would expect union rates to be lower in older patients^14^. A possible reason for this could be compliance issues in the more active and younger patients during their rehabilitation. Unfortunately, we do not have any data on the rehabilitation aspect to study this. In terms of time to union, our regression model found that older patients did have longer time to union as expected. Our study used plain radiographs to determine the union of scaphoid fracture. Although Computed Tomography (CT) scans and Magnetic Resonance Imaging (MRI) may provide better evidence of union^33,34^, it is not routinely used in our clinical setting. Plain wrist radiographs have been found to be adequate and reliable in diagnosing union postoperatively^31,35,36^. In addition, our results apply only to open scaphoid fixations with bone grafts and may not necessarily be valid for cases that were performed without bone grafts or performed through minimally invasive techniques.

## Conclusion

In open fixation of scaphoid with compression screw and bone grafting, our retrospective review has shown that union rate and time to union is comparable whether the screw fixation was augmented with an intracarpal K-wire or not. There are no increased complications associated with the additional K-wire although K-wire removal may be required in some patients after fracture healing. Our audit also found that older age was a significant factor with improved union rate, but increased time to union.
